# Molecular profiling of ctDNA in pancreatic cancer: Opportunities and challenges for clinical application

**DOI:** 10.1016/j.pan.2020.12.017

**Published:** 2021-03

**Authors:** L. Sivapalan, H.M. Kocher, H. Ross-Adams, C. Chelala

**Affiliations:** a Centre for Cancer Biomarkers and Biotherapeutics, Barts Cancer Institute, Queen Mary University of London, EC1M 6BQ, UK; b Centre for Tumour Biology, Barts Cancer Institute, Queen Mary University of London, EC1M 6BQ, UK

**Keywords:** Biomarkers, Cell-free DNA (cfDNA), Circulating tumour DNA (ctDNA), Liquid biopsy, Precision medicine

## Abstract

Pancreatic ductal adenocarcinoma (PDAC) is predicted to become the second leading cause of cancer-related mortality within the next decade, with limited effective treatment options and a dismal long-term prognosis for patients. Genomic profiling has not yet manifested clinical benefits for diagnosis, treatment or prognosis in PDAC, due to the lack of available tissues for sequencing and the confounding effects of low tumour cellularity in many biopsy specimens. Increasing focus is now turning to the use of minimally invasive liquid biopsies to enhance the characterisation of actionable PDAC tumour genomes. Circulating tumour DNA (ctDNA) is the most comprehensively studied liquid biopsy analyte in blood and can provide insight into the molecular profile and biological characteristics of individual PDAC tumours, in real-time and in advance of traditional imaging modalities. This can pave the way for identification of new therapeutic targets, novel risk variants and markers of tumour response, to supplement diagnostic screening and provide enhanced scrutiny in treatment stratification. In the roadmap towards the application of precision medicine for clinical management in PDAC, ctDNA analyses may serve a leading role in streamlining candidate biomarkers for clinical integration. In this review, we highlight recent developments in the use of ctDNA-based liquid biopsies for PDAC and provide new insights into the technical, analytical and biological challenges that must be overcome for this potential to be realised.

## Introduction

Pancreatic ductal adenocarcinoma (PDAC) is the most frequently occurring cancer of the exocrine pancreas and a leading cause of cancer deaths worldwide [1]. PDAC tumours have a propensity for perineural and vascular local growth, in addition to early distant metastasis [2]. This precludes surgical resection for >80% patients, which is currently the only possible curative treatment [2]. Early diagnosis of curable disease remains a significant challenge in primary care, due to the absence of cancer-specific symptoms and the lack of sensitive and specific biomarkers for prospective screening of high-risk populations [3].

Systemic chemotherapy and the more recent use of combinatorial treatments are therefore regarded as the standard of care for the majority of patients who are diagnosed with inoperable disease. However, significant benefits from these therapies have only been observed in small, yet to be characterised groups of patients, and the impact on overall survival rates in PDAC has been marginal (5-year overall survival rate ∼7%, with most survivors seen in the ∼15% of patients with localised, resectable disease) [1]. Resistance to conventional chemotherapies remains a hallmark of PDAC tumours, owing to a complex interplay between genetic and epigenetic alterations, and a highly desmoplastic, hypoxic, hypovascular tumour microenvironment [4]. Together, these factors highlight the need for novel, molecularly-guided strategies to facilitate improved detection, monitoring and treatment of PDAC.

Liquid biopsies are an emerging application of precision medicine, with the potential to inform targeted strategies for early diagnosis, treatment and response monitoring in patients. In this review, we evaluate the clinical significance, technical complexities and biological challenges associated with the most comprehensively studied liquid biopsy analyte in PDAC samples to date, circulating tumour DNA (ctDNA).

## Molecular landscape of PDAC tumours

**Fig. 1:**
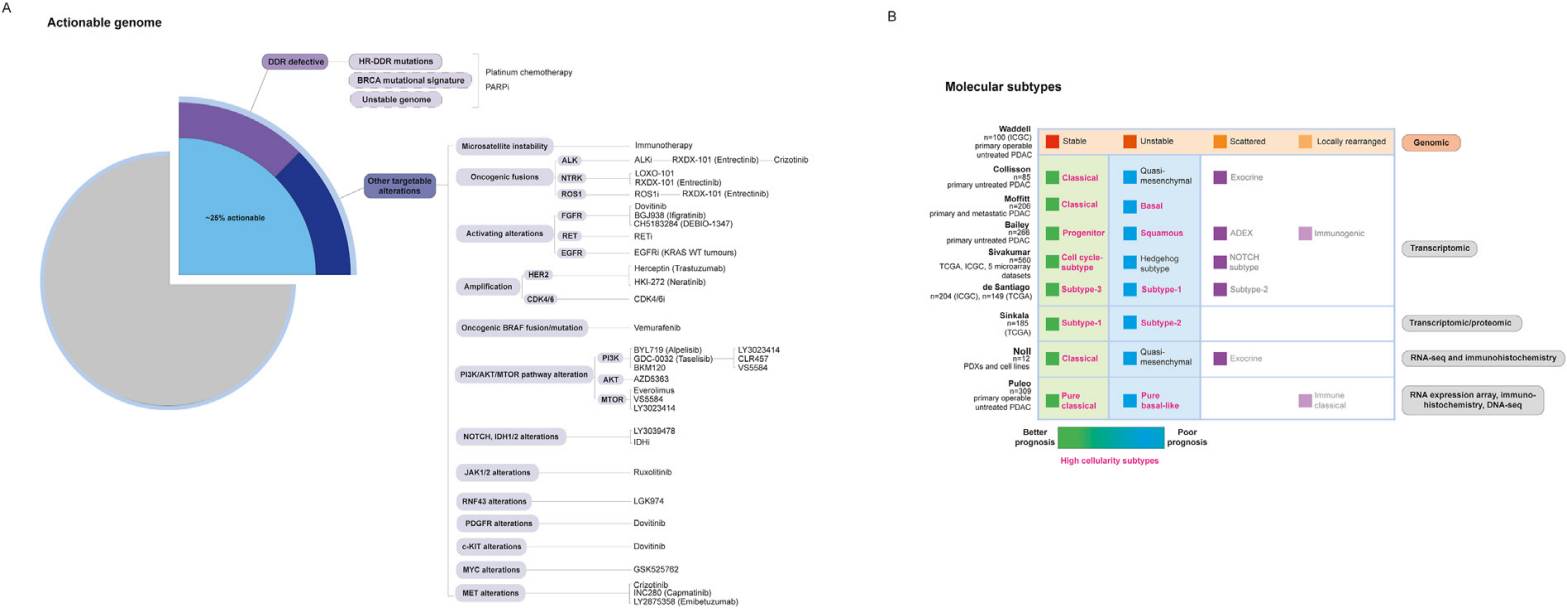
Actionable mutations and molecular subtypes of PDAC tumours. (A) Around 25% of PDAC tumours harbour actionable molecular alterations, for which there is existing strong clinical/pre-clinical evidence of predicted benefit from targeted treatments. Approximately 50% of PDAC tumours with actionable molecular alterations are known to bear mutations within the DNA damage repair pathway. Additional lower-prevalence mutations have also been detected within alternative oncogenic drivers, as shown. (B) Somatic alterations have been studied in combination with chromosomal structural variants or transcriptomic/proteomic profiles to identify underlying molecular subtypes of PDAC tumours. Genomic subtypes have been characterised by Waddell et al. (2015) based on unique patterns of chromosomal structural variation. Several transcriptomic subtypes of PDAC tumours have also been proposed, with most studies converging on the presence of two prognostically relevant tumour subtypes amongst high cellularity samples *(pink)*, of either a classical/progenitor (better prognosis) or basal/squamous phenotype (poorer prognosis). These findings have been reflected in recent integrated subtype classification studies. DDR, DNA damage repair deficiency; HR, homologous recombination; (i), inhibitor.

Genome sequencing of PDAC primary tumour lesions has confirmed the presence of frequent mutations across four key driver genes **(*KRAS, TP53, SMAD4, CDKN2A*)** that are altered in >90% of patients [5,6]. In contrast, somatic variants within alternative driver genes that are commonly targeted for treatment in other cancer types (e.g. *BRAF, KIT*) occur at only a low median prevalence of ≤5% in PDAC, reflecting extensive **inter-tumoural genetic heterogeneity** (Fig. 1a) [5,6]. Efforts to characterise these variations have grouped individual mutations according to molecular mechanisms or biological pathways, in order to constitute more clinically meaningful proportions and to determine tumour subtypes [[7], [8], [9], [10], [11], [12]]. Results from recent subtyping studies, combining the analysis of transcriptomic variation with proteomic and/or immunohistochemistry profiling, have provided growing consensus for the presence of two overarching molecular subtypes of PDAC tumours amongst high-cellularity samples (Fig. 1b) [[13], [14], [15]]. However, clear subtype-specific therapeutic vulnerabilities have not yet been demonstrated for these classifications, despite their established relevance for prognosis (Fig. 1b) [[7], [8], [9], [10], [11]].

These findings highlight several challenges facing tissue-based molecular classifications of PDAC tumours, particularly concerning the **low neoplastic cellularity** of most tumours and the overall lack of resected tumour tissue specimens. This demonstrates the need for alternative sources of tumour analytes that can be sequenced alongside tumour tissues, in order to improve the characterisation of actionable variants within individual patients. Analytes that can be sampled using only minimally invasive methods can allow for genotyping of resectable, and advanced unresectable PDAC tumours that have been vastly understudied to date, due to the unavailability of suitable biopsy specimens [2]. Minimally invasive sampling can also provide new opportunities for longitudinal molecular analysis in PDAC tumours, for the development of integrated tumour monitoring strategies [16,17].

## Sampling and analysis of ctDNA in PDAC

**Cell free DNA (cfDNA)** in peripheral blood comprises a range of extracellular DNA molecules from various sources, including fragments of **circulating tumour DNA (ctDNA)** shed from primary and/or metastatic lesions in patients with cancer [18]. Studies in gastrointestinal (GI) cancers have shown that ctDNA fragments can be isolated from patient plasma and analysed within a clinically meaningful timeframe [19]. Furthermore, molecular analyses of ctDNA and matched tissue biopsy specimens have demonstrated that ctDNA can provide valuable aggregate information on multiple clonal subsets within both primary tumours and metastases in patients, and may provide greater utility for the identification of heterogeneous and clinically relevant tumour subclones, compared to a single-lesion biopsy [[20], [21], [22], [23]].

### Sources of ctDNA in peripheral blood

**Fig. 2:**
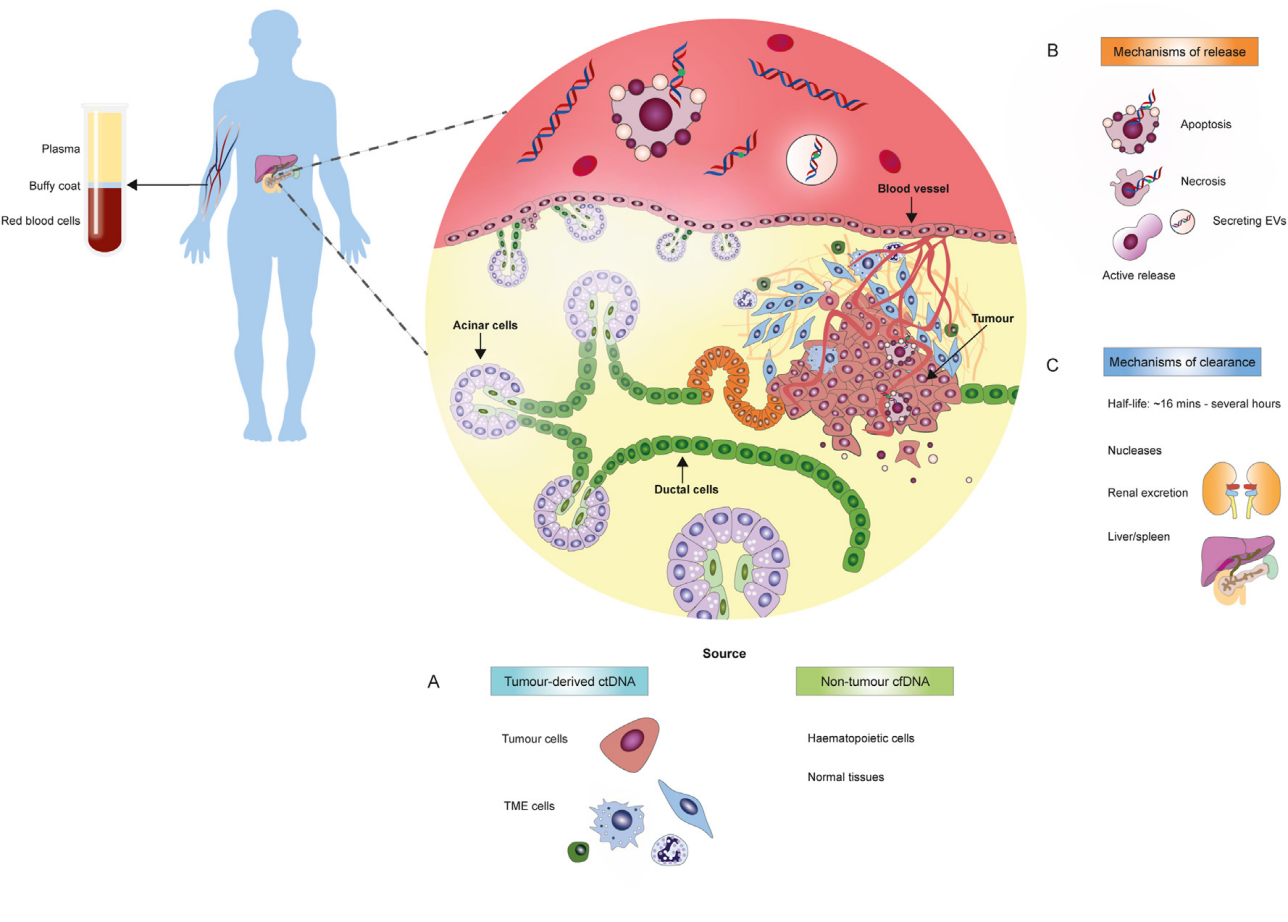
Origins of circulating tumour DNA in the blood. (A) Haematopoietic cells are the predominant source of basal cfDNA levels in both cancer patients and healthy individuals, with fragments bearing distinct epigenetic characteristics consistent with lymphoid and myeloid cells-of-origin. In contrast, tumour lesions comprise a complex mixture of neoplastic cells and cells of the surrounding microenvironment, including stromal cells, immune cells and endothelial cells. These different cell types shed varying levels of tumour-derived ctDNA into the pool of circulating cfDNA throughout tumorigenesis and disease progression. (B) ctDNA can be released into the circulation during tumour cell apoptosis, necrosis or active secretion via extracellular vesicles and/or proliferating tumour cells. **(C)** Mechanisms of ctDNA clearance from the blood are less well understood, but likely include digestion by nucleases, renal uptake or clearance by the liver and spleen. ctDNA, circulating tumour DNA; cfDNA, cell-free DNA; EV, extracellular vesicles; TME, tumour microenvironment.

Circulating tumour DNA is thought to be most commonly released into the circulation during apoptotic tumour cell death (Fig. 2) [24]. However, the release of ctDNA from dying tumour cells can be influenced by tumour type, stage, clonality, replication rates and response to treatment, contributing to significant inter-individual variations between the fractional abundance of ctDNA in blood (Table 1) [25,26]. Moreover, the short half-life of ctDNA fragments in circulation ranges from ∼16 min up to several hours (Fig. 2c) [27,28]. This highlights the importance of carefully defining sample collection times for ctDNA analysis, particularly for post-treatment sampling to monitor tumour response.

**Table 1:** Summary of ctDNA detection and prognostic significance in PDAC.

Study	Biomarker	Source media	Sampling volume	Sampling point	Method of ctDNA detection	Detection technique	Cohort	Extracted cfDNA yield	% ctDNA detection (resectable cases)	% ctDNA detection (unresectable cases)	% ctDNA detection (total cohort)	Conclusions for OS	Conclusions for PFS
Singh et al., 2020	Prognostic	Plasma	–	–	Methylation	Real-time SYBR Green PCR (SPARC, UCHL1, NPTX2, PENK)	n = 65	–	–	–	Methylation indices for all 4 genes higher in PDAC cases, compared to healthy individuals.	Higher ctDNA methylation indices for SPARC and NPTX2 associated with a poorer OS.	–
Strijker et al., 2020	Prognostic	Plasma	4 mL plasma	Pre-treatment	Mutation	Targeted NGS (34 amplicons panel covering KRAS, TP53, NRAS, SMAD4, CDKN2A, PIK3CA, GNAS, BRAF)	n = 58	Median 3.2 ng/μL (range 0.58–23)	–	44.80%	44.80%	Median OS 3.2 months (95% CI 1.6–4.9) vs 8.4 (95% CI 1.6–15.1) months (detection vs undetectable ctDNA, respectively) (0 = 0.005).	–
Sugimori et al., 2020	Prognostic, predictive	Serum	2–3 mL serum	Pre-treatment + during treatment	Mutation	dPCR (KRAS codon 12/13)	n = 45	–	–	51% (baseline)	51% (baseline)	–	Median PFS 248.5 vs 50 days (p < 0.001) for consistent detection vs absence of mutant KRAS ctDNA following chemotherapeutic treatment.
Bernard et al., 2019	Predictive, prognostic	Plasma	1 mL plasma	Pre-treatment + during treatment	Mutation	ddPCR (multiplex assay: KRAS G12D, G12V, G12R, G12C, G12S, G12A, G13D)	n = 194	–	34% (baseline)	53% (baseline)	44% (baseline)	Presence of ctDNA associated with shorter OS (HR, 2.36; 95% CI, 1.16–4.79; p = 0.018), with median OS of 258 vs 440 days (detection vs no detection, respectively). On multivariate analysis, ctDNA detection was a significant predictor of poorer OS in combination with CA19-9 >300 U/mL at pre-treatment baseline sampling (HR, 6.37; 95% CI, 2.36–17.24; p = 0.0003).	Presence of ctDNA associated with significantly shorter PFS (log-rank test: HR, 1.93; 95% CI, 1.15–3.22; p = 0.012). Median PFS of 118 vs 321 days (detection vs no detection).
Eissa et al., 2019	Diagnostic	Plasma	2 mL plasma	Pre-surgery	Methylation	Quantitative methylation-specific PCR (ADAMTS1, BNC1)	n = 39	–	97%	100%	Methylation of either gene in combination panel: 97.3% (sensitivity) + 91.6% (specificity). Individual genes: ADAMTS1 87.2% (sensitivity) + 95.8% (specificity), BNC1 64.1% (sensitivity) + 93.7% (specificity).	–	–
Gall et al., 2019	Prognostic	Plasma	–	Pre-surgery	Mutation	Targeted NGS	n = 16	Mean 63.67 ± 24.37 ng/μL	12.50%	–	12.50%	–	–
Groot et al., 2019	Prognostic	Plasma	40 mL whole blood	Pre-surgery + post-surgery	Mutation	ddPCR (KRAS G12D, G12V, G12R, Q61H)	n = 59	–	49% (pre-surgery)	–	49% (pre-surgery)	Pre-operative ctDNA detection vs absence: median OS 14 months vs median OS not reached (p < 0.001). Post-operative ctDNA detection vs absence: median OS of 17 months vs median OS not yet reached at 30 months (p = 0.011).	Median PFS 8 months vs 19 months (p < 0.001) for pre-surgery ctDNA detection vs absence. Median PFS of 5 months vs 15 months (p < 0.001) for post-surgical ctDNA detection vs. absence. Post-surgical ctDNA detection predicted clinical recurrence (sensitivity 90% (95% CI 74–98%), specificity 88% (95% CI 62–98%)) with median lead time of 84 days.
Lee et al., 2019	Pharmacodynamic, prognostic	Plasma	3.5 mL plasma	Pre-surgery + post-surgery	Mutation	Safe-SeqS (KRAS)	n = 42	–	62% (pre-surgery), 37% (post-surgery)	–	62% (pre-surgery), 37% (post-surgery)	Pre-operative ctDNA detection associated with shorter OS at median follow-up (38.4 months): HR 4.1; p = 0.015. Post-operative ctDNA detection associated with shorter OS: HR 4.0; p = 0.003.	Pre-surgery ctDNA detection associated with significantly shorter PFS at median follow-up (38.4 months): HR 4.1; p = 0.002. ctDNA detection was significant predictor of disease recurrence (HR 6.3; 95% CI 2.4–16.2; p ≤ 0.0001) and death (HR 7.5; 95% CI 2.1–27.7; p = 0.002) during multivariate analyses.
Liu et al., 2019	Diagnostic	Plasma	5–10 mL plasma	Pre-treatment	Mutation/Fragment size	Targeted NGS (62-gene panel)	n = 80	Median 16.2 ng/mL (range 9.3–25.9)	88%	95%	90%	On multivariate analysis, mutant KRAS copy number was a significant predicter of poorer OS (HR: 3.3, 95% CI: 1.1–10.6; p = 0.037)).	–
Mohan et al., 2019	Prognostic	Plasma	–	Pre-treatment	Mutation/Copy number	Targeted NGS (641-gene panel)	n = 55	–	–	62.5% (locally-advanced), 87% (metastatic)	76%	Combined presence of KRAS ctDNA mutations and KRAS copy number gain associated with poorer overall prognosis (median survival 2.5 months, log-rank p-value < 0.0001).	–
Patel et al., 2019	Pharmacodynamic, prognostic	Plasma	10 mL whole blood	Pre-treatment/post-treatment	Mutation	Targeted NGS (54–73 gene panel)	n = 112	5–30 ng total yield	–	–	70%	In univariate analysis, presence of *KRAS* mutations in ctDNA and percentage ctDNA abundance (≥0.6%) associated with poorer OS.	–
Pratt et al., 2019	Diagnostic	Plasma	1–2 mL plasma	Pre-treatment + post-treatment	Mutation	ddPCR	n = 7	Median 3–49 ng/mL	–	86%	86%	–	–
Wang et al., 2019	Diagnostic	Plasma	1–4 mL whole blood	Pre-treatment	Mutation	ddPCR (KRAS codon 12, 13)	n = 95	–	–	–	47.40%	–	–
Watanabe et al., 2019	Pharmacodynamic	Plasma	2 mL plasma	Pre-treatment + post-treatment	Mutation	ddPCR (KRAS G12D, G12V, G12R, Q61H)	n = 78	–	48.70%	71.80%	62.80%	Post-operative emergence of KRAS mutant ctDNA (HR = 54.5, 95% CI: 6.64–447.6, p < 0.001 significant factor for poorer OS. Emergence of KRAS mutant ctDNA (HR = 10.4, 95% CI: 2.95–37.0, p < 0.001) was only significant factor for OS in unresectable patients.	Emergence of KRAS mutant ctDNA within 6 months of chemotherapy significantly associated with poorer PFS (median PFS: 14.9 months versus 4.8 months).
Wei et el. 2019	Predictive	Plasma	–	Pre-treatment + post-treatment	Mutation	Targeted NGS (560-gene panel)	n = 38	Median 28.4 ng/mL	–	–	66%	Poorer prognosis observed in patients with ctDNA MAF >1.5%, compared to patients with <1.5% ctDNA MAF.	–
Berger et al., 2018	Pharmacodynamic, predictive	Plasma	2 mL plasma	Pre-treatment + post-treatment	Mutation	Targeted NGS (TP53, SMAD4, CDKN2A, KRAS, APC, ATM, FBXW7) and ddPCR	n = 20	–	–	80%	80%	–	Combined ctDNA MAFs of KRAS and TP53 during treatment were significantly correlated with PFS (Spearman, r = −0.8609, p = 0.0013).
Cohen et al., 2018	Diagnostic	Plasma	7.5 mL plasma	Pre-surgery	Mutation + proteins	Targeted NGS (16-gene panel)	n = 93	Median 7.54 ng/mL	–	–	72% (mutations + proteins)	–	–
Hellwig et al., 2018	–	Plasma	8 mL plasma	–	Mutation/Fragment size	ddPCR, targeted NGS (128-gene panel)	n = 2 PDAC	20.1 ± 14.5 ng/mL (yield across PDAC, colorectal, melanoma cohorts)	–	–	100%	–	–
Kim et al., 2018	Prognostic	Plasma	1 mL plasma	Pre-treatment + post-treatment	Mutation	ddPCR (multiplex assay: KRAS G12D, G12V, G12R, G12C, G12S, G12A, G13D)	n = 77	Median 427 ng/mL	69%	83% (locally-advanced), 86% (metastatic)	78%	Low (≤41.5%) vs high ((>41.5%) *KRAS* ctDNA MAF associated with OS: 13 vs 8 months. Mutant KRAS ctDNA concentration identified as a prognostic factor for OS (HR 1.97, 95%CI 1.05–3.67).	High (>41.5%) vs low (<41.5%) KRAS ctDNA MAF associated with shorter PFS: 12.6 vs 4.7 months. Mutant KRAS ctDNA concentration identified as a prognostic factor for PFS (HR 2.08, 95%CI 1.20–3.63).
Kruger et al., 2018	Predictive, prognostic	Plasma	–	Pre-treatment + during treatment	Mutation + proteins	dPCR (KRAS)	n = 54	–	–	67%	67%	Presence of KRAS mutant ctDNA and higher pre-treatment levels of CA19-9, CEA and CYFRA 21-1 were significantly correlated with a poorer OS.	Serial measurement of KRAS mutant ctDNA during follow-up was superior to protein-based markers for detection of tumour progression: sensitivity (83%), specificity (100%).
Lapin et al., 2018	Prognostic	Plasma	4 mL (1–2 mL for 8 patients)	Pre-treatment + post-treatment	Fragment size analysis	Fragment size analysis	n = 61	Locally advanced: median 3.26 ng/mL (range 1.16–7.98); Metastatic: median 6.58 ng/mL (range 0.53–1911.63)	–	–	cfDNA fragment size: healthy controls (median 176.5bp, range 168–185bp), locally-advanced PDAC (median 170bp, range 167–173bp), metastatic (median 167bp, range 148–180bp). Fragment sizes significantly larger in healthy controls vs locally-advanced (p = 0.001)/metastatic (p < 0.001) PDAC.	Short pre-treatment cfDNA fragment sizes (≤167 bp) were associated with poorer OS (4.6 months vs 10.5 months; log-rank p = 0.001). Pre-treatment cfDNA levels were independent predicter of poorer OS (HR = 2.236, p = 0.028).	Pre-treatment cfDNA levels were independent predicter of shorter PFS (HR = 3.049, p = 0.005).
Lin et al., 2018	Predictive	Plasma	2 mL plasma	Pre-treatment	Mutation	ddPCR (KRAS)	n = 65	–	–	–	80%	ctDNA detection vs absence: median OS 11.4 months vs 14.3 months (P < 0.001). On multivariate analysis, ctDNA presence identified as independent prognostic factor associated with poorer OS (HR = 3.1, 95% CI: 1.6–4.9, p < 0.001).	–
Mouliere et al., 2018	–	Plasma	2 mL plasma	Pre-treatment + post-treatment	Mutation/Fragment size	Fragment size analysis, sWGS, TAM-Seq, WES	n = 7 PDAC	–	–	–	17% (across low-ctDNA cancers: glioma, renal, bladder, and pancreatic)	–	–
Nakano et al., 2018	Predictive, prognostic	Serum	1–4 mL serum	Pre-surgery + post-surgery	Mutation	PNA clamp PCR (KRAS codons 12, 13)	n = 45	–	24.4% (pre-surgery), 44.4% (post-surgery)	–	24.4% (pre-surgery), 44.4% (post-surgery)	Change in KRAS mutation dynamics (pre-surgery wild-type to post-surgery mutant) significantly associated with poorer OS (HR 9.42, 95%CI 2.02–44.04, p = 0.004)).	–
Park et al., 2018	Diagnostic, predictive	Plasma	2–5 mL plasma	Pre-treatment + post-treatment	Mutation	Targeted NGS (83-gene panel)	n = 17	–	–	–	88.20%	–	–
Perets et al., 2018	Pharmacodynamic, predictive	Plasma	–	During treatment	Mutation	Targeted NGS (KRAS exon 2)	n = 17	–	–	29.40%	29.40%	Mutant KRAS ctDNA detection vs absence: 8 vs. 37.5 months.	–
Riviere et al., 2018	Pharmacodynamic	Plasma	–	–	Mutation	Targeted NGS (68-gene panel, Guardant360)	n = 25	–	–	–	64% (known mutations), 100% (all mutations)	–	–
Shroff et al., 2018	Predictive	Plasma	–	Pre-treatment	Mutation	Targeted NGS (62-gene panel, Foundation Medicine)	n = 16	–	–	69%	69%	–	–
Adamo et al., 2017	Prognostic	Plasma	–	Pre-treatment	Mutation	ddPCR, targeted NGS (50 gene-panel))	n = 26	Median 585 ng/mL (range 120–4180)	17%	40%	35%	Presence of KRAS mutant ctDNA vs absence: 60 vs. 197 days. KRAS mutant ctDNA identified as prognostic factor for OS (HR 2.89 95%CI 1.2–7.3).	–
Ako et al., 2017	Prognostic	Serum, plasma (paired)	1 mL	Post-treatment	Mutation	ddPCR (KRAS G12D, G12V, G12R)	n = 40	17.9 ng/mL (plasma), 129 ng/mL (serum)	–	–	48% (serum), 48% (plasma)	Presence of KRAS G12V alleles in serum or plasma ctDNA associated with poorer OS (p < 0.01).	–
Allenson et al., 2017	Diagnostic	Plasma	0.9–1.5 mL plasma	Pre-surgery + post-surgery	Mutation	ddPCR (multiplex assay: KRAS G12D, G12V, G12R, G12C, G12S, G12A, G13D)	n = 52	–	45.50%	58%	50%	Presence of KRAS mutant ctDNA vs absence (metastatic patients): 115 days vs. 506 days OS (p = 0.107).	–
Cohen et al., 2017	Diagnostic	Plasma	7.5 mL plasma	Pre-surgery	Mutation + proteins	Targeted NGS	n = 221	Median 5.92 ng/mL (range 0.51–121.81)	30%	–	30%	ctDNA detection using combination assay was independent predictor of OS (HR = 1.76, 95% CI 1.10–2.84, p = 0.018, multivariate analyses).	–
Del Re et al., 2017	Pharmacodynamic	Plasma	3 mL plasma	Pre-treatment + during treatment	Mutation	ddPCR (KRAS (G12D, G12V, G12R, G13D))	n = 27	–	–	70% (baseline)	70% (baseline)	Increase vs reduction in KRAS mutant ctDNA abundance at day 15 follow-up (median OS 6.5 vs 11.5 months, p = 0.009).	Increase vs stability/reduction in KRAS mutant ctDNA abundance at day 15 follow-up (median PFS 2.5 vs 7.5 months, p = 0.03).
Henriksen et al., 2017	Prognostic	Plasma	500μL plasma	Pre-treatment	Methylation	Methylation-specific PCR (28-gene panel)	n = 95	–	–	–	–	Decreased 6-month, 1-year and 2-year OS observed for patients with 0–10 hypermethylated genes in ctDNA (73% (95% CI; 61%–82%), 56% (95% CI; 43%–66%).	–
Pishvaian et al., 2017	Prognostic	Plasma	20 mL whole blood	Post-treatment	Mutation	Targeted NGS (68-gene panel, Guardant360)	n = 34 (n = 26 ctDNA analysis)	–	–	–	73%	ctDNA detection vs absence: 11/24 deaths vs. 1/10 deaths (log-rank p = 0.045).	–
Song et al., 2017	Diagnostic	Plasma	–	Pre-treatment + post-treatment	5hmC analysis	5hmC sequencing	n = 7	–	–	–	Up-regulation and down-regulation of 5hmC genes (ZFP36L1, DCXR, GPR21, SLC19A3) in PDAC, compared to healthy controls.	–	–
Van Laethem et al., 2017	Predictive	Plasma	–	During treatment	Mutation	dPCR (KRAS)	n = 60	–	–	65%	65%	Presence vs absence of KRAS mutant ctDNA: median OS 6.6 months vs 18.2 months, respectively.	Presence vs absence of KRAS mutant ctDNA: median PFS 5.3 months vs 8.8 months, respectively.
Vietsch et al., 2017	Pharmacodynamic, predictive	Plasma	200μL plasma	Pre-surgery + post-surgery	Mutation	Targeted NGS	n = 5	–	100% (range 5–12 mutations in 14/56 genes assessed)	–	100%	–	
Berger et al., 2016	Diagnostic	Plasma	2 mL plasma	–	Mutation	ddPCR (GNAS GNAS R201C, R201H, KRAS G12D/G12V)	n = 24	Median 4.22 ng/μL ± 2.501	–	25% (GNAS), 42% (KRAS)	25% (GNAS), 42% (KRAS)	–	–
Brychta et al., 2016	Diagnostic	Plasma	2 mL plasma	Pre-surgery	Mutation	Chip-based dPCR (KRAS codon 12)	n = 50	Median 43.9 ng/mL (range 4.14–250)	35%	–	35%	–	–
Hadano et al., 2016	Prognostic	Plasma	1 mL plasma	Pre-surgery	Mutation	ddPCR (G12D, G12V, G12R)	n = 105	–	–	–	31%	Presence vs absence of KRAS mutant ctDNA: 13.6 vs. 27.6 months. Presence vs absence of KRAS mutant ctDNA identified as prognostic factor for OS (HR 3.2, 95%CI 1.8–5.4).	Presence vs absence of KRAS mutant ctDNA: 6.1 vs. 16.1 months PFS.
Henriksen et al., 2016	Diagnostic	Plasma	500μL plasma	Pre-treatment	Methylation	Methylation-specific PCR (28-gene panel)	n = 95	Median 11.60 ng/mL (range 0.60–957.17)	–	–	Mean number of methylated genes in PDAC cases (8.41 (95% CI 7.62–9.20)) significantly higher than in acute/chronic pancreatitis controls (4.74 (95% CI 4.40–5.08)) (p < 0.001). Combined model (age >65 + hypermethylation frequencies of BMP3, RASSF1A, BNC1, MESTv2, TFPI2, APC, SFRP1 and SFRP2): 76% (sensitivity), 83% (specificity) for PDAC.	–	–
Pietrasz et al., 2016	Prognostic	Plasma	2 mL plasma	Pre-treatment	Mutation	ddPCR (KRAS G12D, V, R), targeted NGS	n = 135	Mean 92 ± 201 ng/mL (resectable 52.5 ± 79.5, unresectable 105.8 ± 227.25)	19%	48%	41%	Presence vs absence of ctDNA: (unresectable patients) 6.5 vs. 19 months OS (log-rank p < 0.001); (resectable patients) 19.3 vs. 32.2 months (p = 0.027). ctDNA detection identified as prognostic factor for OS (HR 1.96, 95%CI 1.2–3.2).	Presence vs absence of ctDNA: (resectable patients): 4.6 vs. 17.6 months (log-rank p = 0.03).
Takai et al., 2015	Predictive	Plasma	2 mL plasma	Pre-treatment	Mutation	ddPCR (KRAS G12D/V/R and G13D), targeted NGS (60-gene panel)	n = 259	Median 20.13 ng/2 mL plasma (stage IV 21.65, stage I-III 17.59)	8.30%	47%	32%	Presence of KRAS mutant ctDNA identified as prognostic factor associated with poorer OS (HR 3.04).	–
Zill et al., 2015	Pharmacodynamic	Plasma	1 mL plasma	Pre-treatment + during treatment	Mutation	Targeted NGS (54-gene panel)	n = 18	–	–	89%	89%	–	–
Bettegowda et al., 2014	Diagnostic	Plasma	2 mL plasma	Pre-treatment	Mutation	dPCR, PCR/ligation, Safe-SeqS	n = 155	–	≥49% (localised)	>80% (metastatic)	≥49% (localised), >80% (metastatic)	–	–

Abbreviations: 5hmC, 5-hydroxymethylcytosine; CP, chronic pancreatitis; ddPCR, droplet digital PCR; NGS, next-generation sequencing; Safe-SeqS, Safe Sequencing System; TAM-Seq, tagged amplicon deep sequencing; sWGS, shallow whole genome sequencing; WES, whole exome sequencing.

### Methods used for ctDNA detection

#### Somatic mutations and copy number alterations

**Fig. 3:**
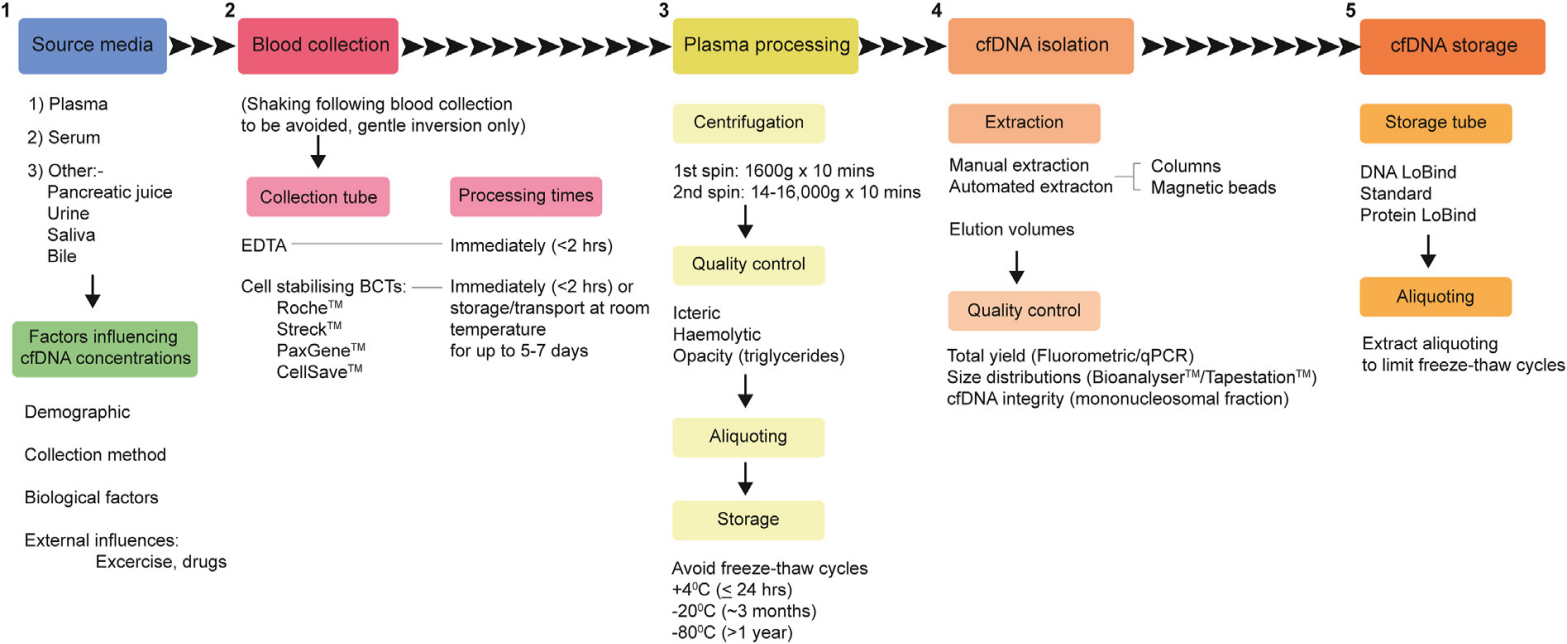
Guidelines for cfDNA isolation and analysis from peripheral blood. Recommended (1) preanalytical considerations and specific application requirements for (2) blood collection, (3) plasma processing, (4) cfDNA isolation and (5) cfDNA storage are shown. Best practice suggests that whole blood samples are processed as soon as possible following blood draw for plasma retrieval, particularly when collected in EDTA tubes. Cell stabilising collection tubes are also commercially available, that maintain sample integrity during transport, or when immediate in-house processing is not possible. During cfDNA extraction, the required concentration of input DNA for downstream applications should be considered when deciding on elution volumes (e.g. to provide the highest possible concentration of ctDNA fragments that are otherwise present at low concentrations in human plasma). Total extracted DNA yields can be measured using fluorometric or PCR-based approaches, and the quality of isolated DNA determined through the analysis of fragment size distributions. qPCR assays can also be used to make complementary assessments of cf-/ctDNA integrity and improve the stringency of sample validations for next-generation sequencing and digital PCR applications. Care should be taken when storing cfDNA samples to limit freeze thaw cycles, which can damage the integrity of fragmented cfDNA. BCT, blood collection tube.

The ability to detect tumour-derived somatic mutations in plasma depends on the number of isolated ctDNA template molecules, which in turn dictates the allelic fractions of mutations present. This can be further affected by the pre-analytical effects of different plasma processing protocols on the isolation efficiency and resulting mutant allele fractions (MAFs) of ctDNA (Fig. 3) [29,30]. Whilst the fractional abundances of ctDNA can exceed 10% of total cfDNA in patients with advanced cancers, levels are typically much lower (≤1%) in patients with early or **minimal residual disease (MRD)**, presenting a significantly greater challenge for detection efforts [26,31,32]. Furthermore, pan-cancer comparisons have demonstrated that plasma samples from patients with PDAC generally harbour lower ctDNA burdens, compared to other solid tumour types, including breast and ovarian cancer [26,33]. This highlights a profound obstacle for accurate ctDNA detection in PDAC plasma samples, particularly from patients with early-stage disease, as rare mutant molecules are more likely to be affected by stochastic sampling variations [34]. These effects are likely to explain the low concordance between reported ctDNA detection rates across PDAC studies to date (Table 1) [34]. Tumour-specific amplifications and deletions can also be identified through shallow (∼0.1X coverage) whole genome sequencing (sWGS) of matched ctDNA and germline DNA. However, the limit of detection of sWGS approaches for ctDNA is ∼5–10%, which can severely restrict sensitivity for profiling **early disease** in PDAC [35,36].

#### Methylation profiling

Although methylation profiling has not been performed extensively throughout PDAC ctDNA studies, this approach is gaining traction for its ability to provide complementary information to mutation analyses, particularly in the context of early disease [[37], [38], [39], [40], [41], [42], [43]]. Lehmann-Werman et al. (2016) identified 2 CpG sites (within the *CUX2* and *REG1A* loci), that were differentially unmethylated in the exocrine pancreas compared to the endocrine pancreas and other tissues [38]. Forty-eight percent of patients with pancreatic cancer were found to have circulating levels of unmethylated exocrine pancreas markers above background (highest signal observed in healthy controls), with stronger signals observed in patients with stage III/IV disease [38]. Differences between circulating methylation profiles were also demonstrated between patients with PDAC and chronic pancreatitis, reflecting the potential for combined approaches targeting methylation and mutation profiles to improve both the sensitivity and specificity for ctDNA detection [38]. In addition to these findings, Eissa et al. (2019) recently reported that the methylation status of the genes *ADAMTS1* and *BNC1* may be a promising ctDNA biomarker of early-stage PDAC, with high sensitivity (94.8%) and specificity (91.6%) for detection of localised (stage I and II) disease [39].

#### Biological characteristics

**Fig. 4:**
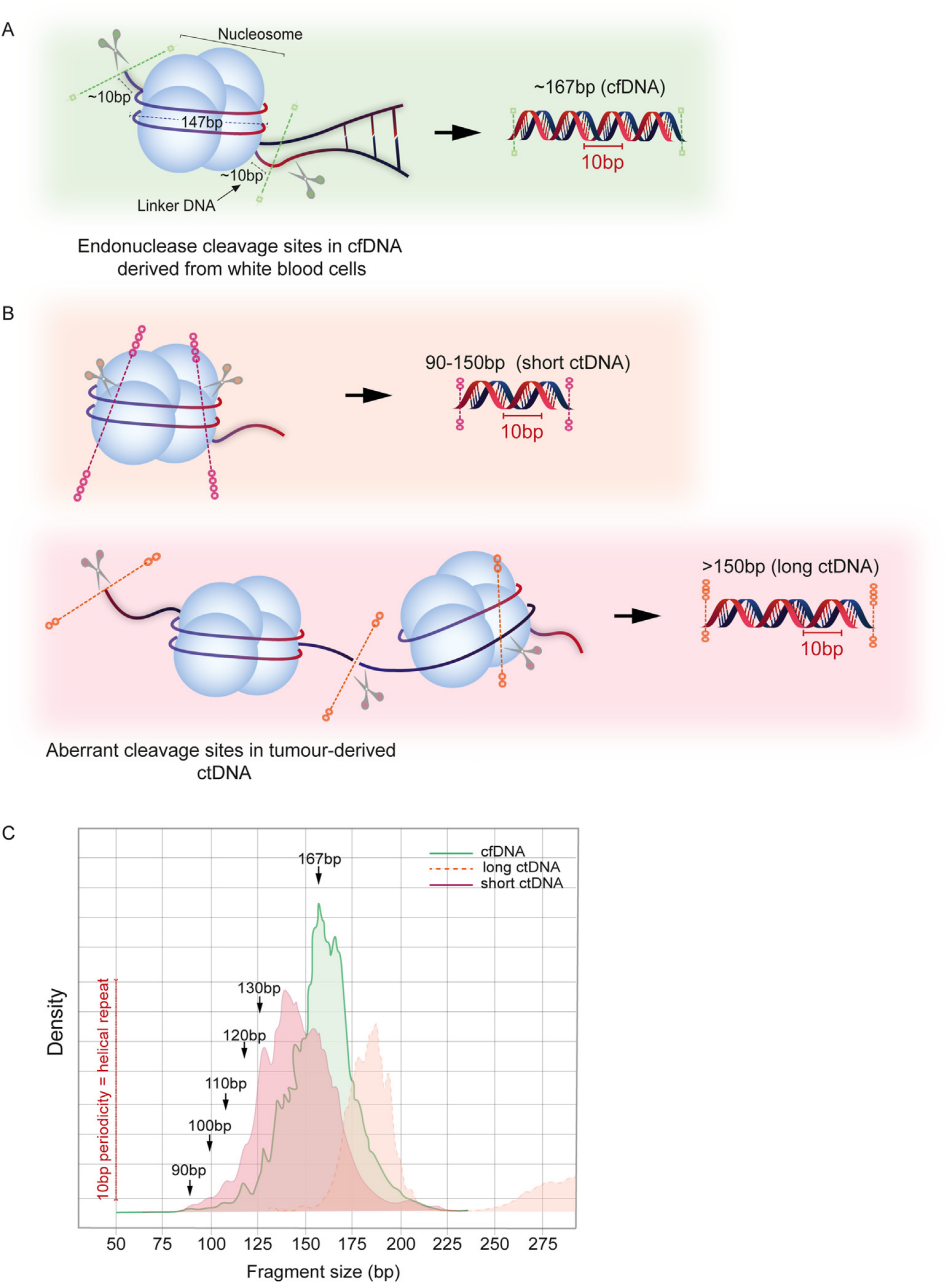
Cell-free DNA fragmentation patterns in PDAC. (A) Cell free DNA molecules display a characteristic modal fragment size ∼167bp; 147bp of DNA are wrapped around a nucleosome with a 10bp periodicity corresponding to the helical pitch of DNA on the nucleosome core, and 20bp of linker DNA constitute the remainder of cfDNA fragments. Although DNA within the nucleosome core is protected from endonuclease activity, regions of linker DNA remain vulnerable to digestion, leading to small variations in this modal fragment size between cfDNA samples, which can be explained by varying linker lengths. (B) In contrast, ctDNA fragmentation profiles have been shown to be more variable than non-tumour cfDNA, as tumour cell necrosis and mechanisms of active release can also contribute to overall levels of tumour-derived ctDNA fragments in blood. Mutant ctDNA fragments in PDAC have been detected at both short (≤150bp) and long (>150bp) fragment sizes, with varying degrees of enrichment observed within each size fraction. (C) Patterns of cfDNA and ctDNA cleavage are evidenced in fragment size distribution profiles, as shown in (B).

Furthermore, several studies have provided proof-of-principle for the combined analysis of genome-wide ctDNA fragmentation patterns with mutation profiles, to improve the overall sensitivity for ctDNA detection [21]. Mutant ctDNA in PDAC has been detected in both short (≤150bp) and long (>150bp) fragments, using size selection and/or profiling methods in combination with bioinformatic algorithms to determine the degree of ctDNA enrichment (Fig. 4) [21,33,44,45]. However, the proportion of short ctDNA fragments is known to be lower in PDAC compared to other solid tumour types, suggesting tissue-specific differences between ctDNA cleavage and fragmentation patterns [33]. In contrast, individual studies have each differed in their definition of ‘long’ ctDNA fragments (e.g. Mouliere et al. (≥320bp) vs Christiano et al. (151-220bp)), rendering accurate cross-cohort and study comparisons difficult [21,33]. Therefore, the validity of approaches to combine the analysis of ctDNA fragmentation patterns with mutation profiles requires rigorous testing in large PDAC sample cohorts to evaluate potential for clinical applicability.

### Techniques for ctDNA analysis

#### Next-generation sequencing/digital PCR

Numerous techniques for ctDNA analysis have been evaluated to date. Of these, **targeted gene sequencing** and **droplet digital PCR (ddPCR)**, have been the most widely explored [16,31,[46], [47], [48]]. In contrast, untargeted methods of detection, such as **whole exome sequencing (WES)**, have not been extensively used in PDAC samples, despite the advantages for identification and tracking of novel genetic changes acquired during treatment and without prior information about individual tumour genomes [[49], [50], [51]]. This is mainly due to high costs and lower overall sensitivities of genome-wide sequencing methods. A way forward is the use of sWGS to estimate ctDNA copy number and fractional abundances in plasma samples prior to WES, to ensure that only samples with a sufficiently high tumour fraction are used for WES profiling [52]. Studies in neuroblastoma and lung cancer have also combined analytical pipelines with various *in silico* error correction approaches to minimise false positive results in exome-wide ctDNA sequencing data, and are promising in the context of PDAC [49,51]. Such strategies for broad genomic profiling offer the potential to circumvent the limitations associated with high levels of inter-tumoural genetic heterogeneity in PDAC, by enabling patient-specific analysis of the landscape of mutated genes in ctDNA. These methods can be applied to the study of both resectable and unresectable patients, in cases where access to matched tumour tissues is limited. The development of optimised analytical pipelines that can enable mutation calling of low frequency ctDNA variants, and distnguish these from an increase in false positive calls and/or sequencing artefacts in WES data will be essential for the successsful application of broad genomic profiling in PDAC samples with a low ctDNA burden.

**Fig. 5:**
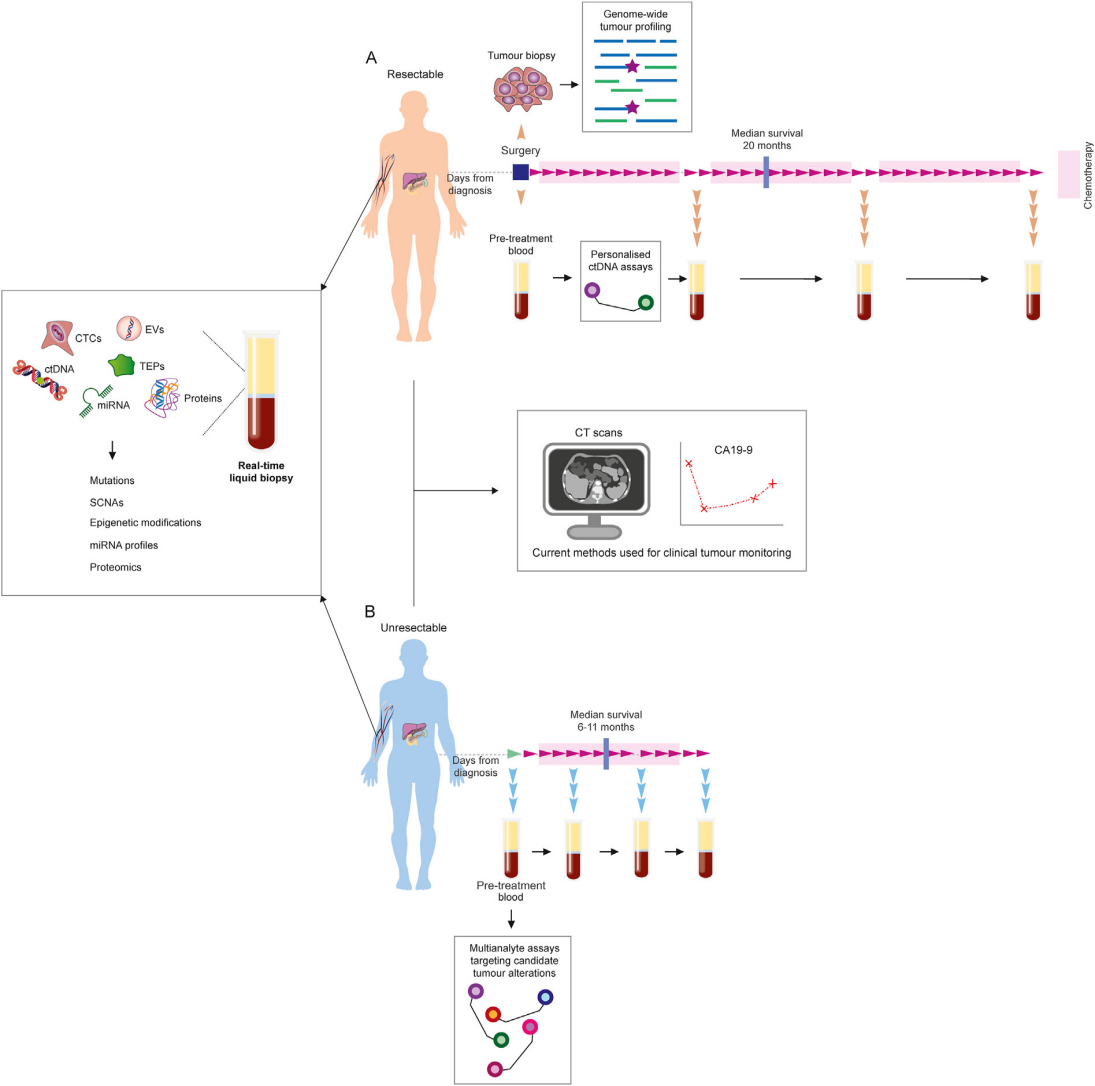
Longitudinal monitoring of PDAC tumours using multianalyte liquid biopsies. (A) Longitudinal monitoring of tumour response to adjuvant treatment, or disease progression following surgical primary tumour resection, is currently performed using routine imaging and by tracking levels of the tumour marker CA19-9 in blood. These measurements can be complemented using liquid biopsy approaches. In resectable cases with matched tumour biopsies available for genomic profiling, personalised assays can be developed to enhance the sensitivity for detection and tracking of rare mutant molecules in blood. (B) New and effective means of monitoring treatment response in patients with unresectable PDAC remains of significant clinical need. In the absence of resected tissue samples for tumour genotyping, multi-analyte liquid biopsy approaches, combining ctDNA and/or CTC detection with the analysis of miRNA and proteins in blood, present an alternative method to enhance the sensitivity for detection of tumour molecules. Unresectable patients also typically have a greater tumour burden and increased levels of circulating tumour molecules in peripheral blood, compared to patients with earlier stages of resectable disease, making them suitable candidates for combined orthogonal sampling and multi-marker profiling. CTC, circulating tumour cell; miRNA, microRNA, SCNA, somatic copy number alterations; TEP, tumour educated platelets.

In contrast, targeted deep sequencing (>10,000X) can detect ctDNA mutations with MAFs as low as <0.2% [[53], [54], [55]]. Similarly, digital PCR platforms can be applied to the analysis of known ctDNA mutations at <0.1% MAFs [56]. The improved sensitivities provided by targeted detection methods highlights their particular utility for the identification and personalised monitoring of rare mutant ctDNA molecules in the MRD setting (Fig. 5). Such personalised ctDNA monitoring strategies, combining genomic profiling of tumour tissues with targeted deep sequencing or ddPCR detection of tumour-derived ctDNA mutations, have been trialled across several solid tumour types, including breast and renal cancer, providing clinical value in select patients [57,58]. However, personalised ctDNA profiling can be costly, and potential applications of this approach in PDAC will be limited to the minority of resectable cases with suitable and substantial primary tumour tissues available for sequencing.

As such, most gene panels that have been tested for clinical utility in PDAC samples have only targeted hotspot regions within the ***KRAS* proto-oncogene**, owing to the prevalence (>90%) of *KRAS* gene mutations in PDAC tumour tissues (Table 1) [17,59,60]. However, mutant *KRAS* detection rates have varied significantly across PDAC ctDNA studies (Table 1). This is likely to be the result of a combination of sampling variation, varying sensitivities of platforms used for ctDNA detection and inter-patient differences between ctDNA fractions in blood plasma. Despite this variability in detection rates, the prognostic relevance of *KRAS* mutation states in PDAC plasma ctDNA samples has been widely shown [59,61], highlighting a need for further investigation into the molecular determinants of these findings. Towards this end, oncogenic dosage gain and variation of mutant *KRAS* has recently been shown to play a critical role in PDAC biology by driving early tumorigenesis and phenotypic diversification [62]. An increase in gene dosage of mutant *KRAS* can result from copy number gains of *KRAS* itself or alternative oncogenic amplifications (*MYC, YAP1, NFκB2*) in combination with heterozygous *KRAS* mutations [62]. These events are associated with high tumour metastatic potential and poor overall clinical outcomes in patients [62]. Oncogenic gains and mutant *KRAS* dosages are further associated with different combinations of secondary hallmark tumour suppressor alterations (*CDKN2A, TGFβ* pathway, *TP53*), which can be evaluated in ctDNA through mutation and copy number profiling [62]. The established prognostic utility of this biomarker indicates promise for the combined analysis of *KRAS* mutations and copy number alterations targeting highlighted key genes in PDAC ctDNA samples, with early studies having already demonstrated clinical potential [60]. Follow-up investigations in large PDAC sample cohorts will be essential to further assess biomarker performance against the accuracy of prognosis.

#### Bioinformatic analysis of ctDNA sequencing data

Numerous bioinformatic pipelines have been developed for the analysis of ctDNA sequencing data. Each approach has differed in the application of error-correction or mitigation methods used to amplify mutant signals in low input samples. Low DNA inputs for sequencing can be compensated for by increasing the number of targets that are interrogated in each sample and establishing a threshold to classify samples as positive, or by increasing the per-sample sequencing depth. However, increasing the depth of sequencing in low-input ctDNA libraries can cause a proportional increase in PCR duplication rates, which can result in a high number of redundant reads within final datasets [63]. This can be exacerbated by the narrow size distribution profile of cf-/ctDNA fragments, which can further lower the complexity of final sequencing libraries [63,64]. These duplicates are normally marked using tools such as Picard MarkDuplicates or Samtools rmdup, and are excluded during variant calling [63]. Whilst effective for the analysis of genomic DNA samples, de-duplication using these tools is not advised for the analysis of fragmented DNA, as numerous sequencing reads originating from distinct fragments of ctDNA may coincidentally share identical mapping coordinates [63]. Marking these reads as duplicates could lead to the simultaneous loss of both true PCR duplicates and genuine ctDNA reads of interest. The resulting decrease in overall sensitivity can have a profound impact on ctDNA detection, particularly in cancers with a low ctDNA burden (such as PDAC), or when profiling early disease or MRD. These effects can be mitigated by attaching unique molecular indexes (UMIs) to each DNA molecule during library preparation, thus identifying PCR duplicates and sequencing errors to be excluded from subsequent analyses [65,66].

Artefacts introduced during library preparation can also significantly affect analytical sensitivity during ctDNA sequencing, particularly at low MAFs ≤0.1% [67]. For example, the use of hybridisation capture baits for enrichment of target genomic regions during exome or gene panel sequencing has been shown to introduce 8-oxoguanine artefacts in sequences at C > A bases, as a result of *ex vivo* oxidative damage [67]. It is vital that appropriate *in silico* error correction methods (e.g. OxoG3 filter, Broad Institute) are applied to minimise the influence of these artefacts on the accuracy of ctDNA detection [67].

## Evaluating the clinical potential of ctDNA in PDAC

Recent advances have demonstrated opportunities for the clinical use of ctDNA fragments as prognostic, diagnostic and pharmacodynamic biomarkers for PDAC, and have shown utility for the identification of therapeutically targetable molecular alterations within individual patients. These findings have provided a foundation for larger prospective studies to assess the clinical benefit of ctDNA analysis for patient management in PDAC.

### Prognostic biomarker

The prognostic relevance of ctDNA detection in patients with PDAC has been demonstrated across several recent studies [59,61,[68], [69], [70], [71], [72], [73], [74], [75]]. Hadano et al. (2016) used ddPCR to detect *KRAS* codon 12 mutations in ctDNA from 105 patients with resectable PDAC [59]. No significant associations were observed between mutant *KRAS* status in tumour tissues and overall survival in patients [59]. In contrast, the presence of detectable *KRAS* mutant alleles within ctDNA was associated with a significantly poorer prognosis, according to both disease-free and overall survival analyses (Table 1) [59]. In a similar study, Pietrasz et al. (2017) used targeted sequencing in combination with ddPCR, for the detection and validation of known PDAC driver mutations in ctDNA from prospectively sampled patients [61]. The presence of *KRAS* mutant ctDNA was shown to be strongly correlated with tumour grade and was independently associated with a poorer overall survival in patients [61]. These findings suggest that ctDNA abundance may be influenced by the biological characteristics of individual PDAC tumour lesions, in addition to overall disease burden. Recently, studies have also demonstrated a significant correlation between the detection of mutant *KRAS* ctDNA at pre-treatment sampling and the presence of liver metastases in patients, indicating potential tissue-specific patterns between ctDNA release from primary and metastatic lesions, which warrant further assessment [20].

### Diagnostic biomarker

The analysis of ctDNA can also provide a useful tool for the early diagnosis of PDAC tumours, when surgical resection is most likely to improve survival [31,32,45,[76], [77], [78], [79], [80]]. In a sample cohort of 221 resectable (stage I-II) PDAC patients*, KRAS* mutant ctDNA was detected in 30% of cases, with 94% of mutations present within codon 12 and a further 6% within codon 61 [31]. However, the number of ctDNA template molecules detected in individual patients remained low across the cohort, with 38% of patients harbouring fewer than 2 mutant templates per mL plasma [31]. More recently, the same group reported on the development of a pan-cancer multi-analyte screening test, called CancerSEEK, capable of detecting somatic mutations across 16 genes in ctDNA (including *KRAS*), and quantifying 8 cancer-associated proteins (carbohydrate antigen 125 (CA-125), CA19-9, CEA, HGF, myeloperoxidase, prolactin, OPN, tissue inhibitor of metalloproteinases 1 (TIMP-1)) in blood [32]. The test was used in 1005 patients previously diagnosed with stage I-III colorectal, breast, gastric, liver, oesophageal, ovarian and pancreatic cancers, with sensitivities of detection that ranged from 98% in ovarian cancer to ∼70% in pancreatic cancer (Table 1) [32]. The application of a supervised machine-learning algorithm to the multi-analyte data was able to accurately predict the location of cancer in ∼80% of pancreatic cancer patients with a positive CancerSEEK test [32]. Whilst these data provide the practical framework for a pan-cancer multi-analyte blood test, considerable improvements in sensitivity are required for the implementation of ctDNA-based diagnostic screening for sporadic PDAC [32]. All individuals included in the study (including PDAC cases) had been already diagnosed with known, in most cases symptomatic, cancers [32]. Whilst the median sensitivity for detection of stage II-III cancers was 73–78%, it was only 43% for stage I disease [32]. These values are likely to be considerably lower in a true screening setting with a lower prevalence of advanced disease, with a further reduction in specificity from the prevalence of cases amongst a larger proportion of non-cancer patients [32].

### Pharmacodynamic biomarker

Longitudinal ctDNA monitoring has not been performed extensively in PDAC cases, owing to the short patient survival times and difficulties in maintaining regular serial blood sample collections outside of an established clinical trial setting. Therefore, CA19-9 remains the only blood biomarker currently used in clinical management for monitoring responses to treatment in patients [81]. However, it performs poorly in detecting small tumours, and cannot be used in non-secreting, Lewis AB- patients (∼10% of all PDAC cases) [81]. Del Re et al. (2017) observed a significantly poorer survival in patients who displayed an increase in mutant *KRAS* ctDNA abundance at 15 days of post-treatment follow-up, compared to patients who displayed either a decrease or stabilisation of detectable ctDNA levels (Table 1) [82]. Radiological evaluation two months after treatment revealed clinical disease progression in all patients who displayed an increase in ctDNA concentration at day 15, indicating the potential for ctDNA profiling to enable early detection of progressive disease [82]. Similarly, Watanabe et al. (2019) demonstrated that the presence/emergence of *KRAS* mutant ctDNA within one year of surgical primary tumour resection was associated with a significantly poorer overall survival in PDAC patients (Table 1), which was not observed for CA19-9 [17]. Comparable findings were observed in unresectable chemotherapy-naïve cases sampled following first-line treatment, highlighting the potential of longitudinal ctDNA tracking for monitoring therapeutic responses in both early and advanced disease settings (Table 1) [17]. These encouraging early results provide a platform for further investigations into the landscape of actionable mutations in PDAC ctDNA samples, and how these may be influenced by different clinical treatments (e.g chemotherapy vs chemoradiotherapy) [17,[82], [83], [84], [85], [86]]. This will be essential to circumvent the limitations associated with variable detection rates of mutant *KRAS* in ctDNA and extend the applications of longitudinal ctDNA monitoring as a pharmacodynamic biomarker for use in larger numbers of patients with PDAC.

### Predictive biomarker

Deep sequencing of PDAC ctDNA samples has also been used to identify high-confidence tumour mutations within therapeutically targetable genes in patients; although to date this approach has been limited to patients with high ctDNA levels [55,83,[87], [88], [89], [90], [91], [92]]. Zill et al. (2015) conducted a prospective analysis of ctDNA samples from patients with advanced pancreaticobiliary tumours, including 18 PDAC patients, using an NGS approach targeting a custom 54-gene panel, without *a priori* knowledge of individual tumour genotypes [88]. Multiple clinically meaningful ctDNA alterations were detected in patients, for whom tissue sequencing was not possible due to insufficient quantities of suitable material [88]. Predictive mutations detected in ctDNA from this cohort included a canonical activating *EGFR* exon 19 deletion, which was detected in the blood of a patient with PDAC 7 months prior to identification during a repeat clinical biopsy [88]. The emergence of the deletion variant during initial treatment with FOLFIRINOX followed by Capecitabine-Oxaliplatin, prompted a switch in treatment to the *EGFR* inhibitor Erlotinib, which coincided with an improvement in patient response [88]. Similarly, Takai et al. (2015) used a custom 60-gene panel on patients with ≥1% *KRAS* MAF in plasma [47]. This led to the identification of ctDNA mutations within several therapeutically targetable genes (including *ATM, EGFR, MAP2K4* and *PIK3CA*) in 14 of 48 patients tested [67]. Notably, individual mutations occurred at a low prevalence in patients (n ≤ 5), reflecting the extent of inter-tumoural genetic heterogeneity previously characterised in PDAC tumour tissues [47].

## Challenges to be overcome

### Need for more precise determination of ctDNA release mechanisms

Several key challenges remain to be overcome, in order to improve the efficiency of ctDNA detection in PDAC and enable clinical implementation. The first is to improve understanding of the mechanisms governing the release of tumour-derived analytes into circulation. This will be essential to determine the kinetics of tumour shedding in PDAC and other cancers. Tumour cell death through apoptosis has long been considered as the most likely origin of ctDNA [33]. However, direct correlations between tumour apoptotic indices and fractional abundances of ctDNA are yet to be proven. Recent studies have suggested that changes within ctDNA dynamics may also be associated with actively proliferating tumour cells [25]. Abbosh et al. (2017) reported a positive correlation between ctDNA detection in patients with non-small-cell lung carcinoma (NSCLC), 2-[^18^F] fluoro-2-deoxyglucose (FDG) avidity on PET imaging and tumour Ki67 proliferation indices, all of which were markers of a poorer overall prognosis [25]. In addition to individual tumour growth and proliferation rates, it is unknown whether different primary tumour or metastatic clones shed ctDNA homogeneously. Further study into the factors governing ctDNA release from spatially distinct tumour clones, and the extent to which they influence overall ctDNA detection rates in PDAC and other cancers, is required.

### Standardisation across pre-analytical workflows

Standardisation across pre-analytical workflows is further required to reduce the variability between ctDNA detection rates in PDAC and improve inter-lab concordance. This need for unified guidance on the handling, documentation and processing of blood samples for circulating DNA isolation was addressed by the International Organisation for Standardisation (ISO), that recently published a set of standards for the appropriate processing of blood samples [93]. To facilitate routine ctDNA testing within a clinical setting, up-to-date validations and external quality assurances of these protocols are required. These have been the recent focus of several international consortia efforts, including CANCER-ID (https://www.cancer-id.eu/) and BloodPAC (https://www.bloodpac.org/).

### Technical limitations to ctDNA detection

Limited starting material can present a considerable technical limitation for the detection of rare tumour-derived markers in blood [31]. The likelihood of capturing rare PDAC ctDNA molecules during blood draw and subsequently incorporating them into a final sequencing library can be directly influenced by the overall concentration of mutant fragments that are present in a patient’s blood. Across most commonly used plasma volumes (∼1–4 mL), the accurate sampling of rare mutant ctDNA alleles, present at MAFs of 0.1% or even 0.01% (Table 1), can therefore represent a physical limitation to ctDNA detection, particularly during early disease or MRD [25].

### Biological challenges to ctDNA detection

Several confounding biological factors can also influence the accuracy of ctDNA detection from blood samples, posing significant challenges for clinical implementation. Firstly, the fractional abundance of ctDNA in blood can be directly influenced by tumour volumes and the number of ctDNA-releasing cells [25,48]. Findings from the TRACERx study revealed that radiological primary tumour volumes in patients with NSCLC were correlated with mean plasma MAFs of clonal ctDNA variants [25]. Based these results, a ctDNA MAF of 0.1% corresponded to an estimated tumour volume of 10 cm^3^ and a MAF of 1.4% with a tumour volume of 100 cm^3^ [25]. A tumour of volume 1 cm^3^ was predicted to correspond to a mean plasma clonal MAF of 0.008% [25]. This presents a direct challenge for the detection of ctDNA fragments released from PDAC lesions, which typically range from ≤2 cm to ∼5 cm in diameter [96,97]. The tumour microenvironment in PDAC can further impair the release of ctDNA fragments into the circulation. PDAC tumours are characterised by extensive desmoplasia, which results in elevated intra-tumoural pressure and hypovascularity [4]. Coupled with a reciprocally low neoplastic cellularity, these features present a rigid barrier to the release of ctDNA fragments into the blood, underpinning the low ctDNA burdens typically observed in patients [4].

Furthermore, the presence of a genuine (non-artefactual) biological mutation in plasma may not necessarily be specific for a population of tumour cells. Both lymphoid and myeloid cells of the haematopoietic lineage are known to accumulate somatic mutations during ageing, which can cause false positive genotype results in plasma [94,95]. Whilst the majority of random mutations acquired during the division of haematopoietic stem/progenitor cells do not have a functional impact, mutations within certain cancer driver genes may confer selective fitness advantages, such as self-renewal or proliferation, which can lead to the clonal expansion of affected cell populations [94,95]. Mutations that accumulate under such circumstances are a form of somatic mosaicism, termed **clonal haematopoiesis of indeterminate potential (CHIP)**, which can confound the interpretation of tumour-derived (ctDNA) variants in blood [18]. Studies show that 5–6% of individuals over the age of 70 years carry somatic driver mutations associated with haematological neoplasia, including low level *BCR-ABL* fusions and oncogenic *BCL2* translocations [94,95]. Therefore, matched plasma-buffy coat sampling is imperative during ctDNA sequencing, in order to identify and effectively filter out CHIP-associated mutations from peripheral blood leucocytes, which could otherwise contribute to false positive ctDNA detection rates [18].

## Future directions

Over recent years, ctDNA testing has been increasingly incorporated into clinical trials for GI cancers, including PDAC, highlighting important progress towards clinical use. The majority of these trials have focussed on the utility of ctDNA-based patient stratification towards targeted therapies. In the recent TARGET programme, patients with advanced cancers were matched to appropriate early phase clinical trials based on the analysis of ctDNA mutations within a 641-cancer gene panel assay [19]. Overall, 41 out of 100 patients were found to harbour actionable ctDNA mutations, of which 11 went on to receive matched therapies with evidence of stable disease or partial response [19]. Interim results from the GOZILA study in patients with metastatic colorectal cancers (CRC), further showed that ctDNA analysis can be equally as informative as tumour tissues, for the identification of patients with *HERC2*-amplified metastatic CRC, who can benefit from dual HERC2-targeted therapy (trastuzumab and pertuzumab combination) [98]. More recently, the study also reported on extended findings across patients with multiple advanced GI cancers, including PDAC [99]. Several clinically actionable variants were detectable in ctDNA from PDAC patients, including pathogenic germline *BRCA* mutations for which polyadenosine-diphosphate-ribose polymerase (PARP) inhibitor therapy is indicated [99].

However, growing indications suggest that the successful application of a single marker or blood-derived analyte for accurate longitudinal monitoring of early stage PDAC tumours, or unresectable cases where there is no prior information about tumour molecular profiles, is unlikely to yield sufficient sensitivity or specificity for clinical use (Fig. 5) [31,32]. In such cases, clinically useful biomarkers for tumour monitoring may only be identified by using integrated detection strategies to supplement mutation data from ctDNA with information on somatic copy number alterations, methylation profiles or gene/protein expression, from alternative sources of blood-derived analytes (Fig. 5) [31,32]. Combined multi-analyte strategies can also present significant advantages for tumour profiling in cancers with high levels of inter-tumoural molecular heterogeneity, such as PDAC, by reducing the effects of sampling variation on overall detection rates [34]. Recent studies have highlighted promising exosome-derived biomarkers for use in future combined strategies, including the cell surface proteoglycan, GPC1, which has been shown to be specifically enriched on PDAC tumour-derived exosomes [100]. Such combined platforms will need to be tested and validated across large-scale multi-centre cohorts, with sufficient sample numbers to establish clinical validity and utility.

## Conclusions

As the most widely studied liquid biopsy analyte in peripheral blood, ctDNA has been investigated for a variety of research and clinical applications. Studies have demonstrated its utility for complementary analysis of tumour genomic profiles and highlighted potential to improve the characterisation of actionable PDAC variants. The clinical relevance of ctDNA profiling in PDAC has also been shown, indicating promise for future uses in early detection, treatment monitoring and the prediction of prognosis in patients. However, the extent of inter-tumoural molecular heterogeneity in PDAC suggests that the clinical uptake of liquid biopsy testing may require greater sensitivity and specificity than that provided by single-analyte approaches targeting ctDNA alone. Towards this end, multi-analyte strategies targeting a range of tumour-derived molecules may represent the future of liquid biopsies for clinical use, with the potential to extend the analytical validity and clinical utility of single-analyte tests. Such methods require rigorous testing in large-scale prospective studies to determine the clinical validity of liquid biopsy profiling for the management of PDAC in patients.
